# Supplementary material for: Transcriptomic Profiling of Adult-Onset Asthma Related to Damp and Moldy Buildings and Idiopathic Environmental Intolerance
Source: Int J Mol Sci. 2021 Oct 1;22(19):10679. doi: 10.3390/ijms221910679 (PMC8508786; doi:10.3390/ijms221910679)
Supplement: Supplementary file 1 [file ijms-22-10679-s001.zip › Suojalehto_SYMBI_Supplement_30.9.pdf]

ONLINE DATA SUPPLEMENT FOR

Suojalehto, Ndika, et al. Transcriptomic profiling of adult-onset asthma related to damp and moldy buildings and idiopathic environmental intolerance

Clinical assessment

— *Asthma medication and severity*

The inhaled steroid dose and the intensity of asthma treatment in asthma patients was graded according to the Global Initiative for Asthma (GINA) guidelines (GINA 2018[1]). The definition of severe asthma was adapted from ERS/ATS criteria[2] and required high-level treatment (GINA treatment step 4-5) together with one of the criteria indicating uncontrolled asthma: poor symptom control, two or more severe exacerbations in the previous year or airflow obstruction[3].

— *Quick Environmental Exposure and Sensitivity Inventory (QEESI) questionnaire*

To gauge IEI we used Chemical intolerances, Symtoms and Life impact scales of the Quick Environmental Exposure and Sensitivity Inventory (QEESI)[4]. Chemical intolerance scale refers to what extent certain odors, symptoms scale assesses symptoms of different organ systems and exposures makes one sick and life impact scale to what extent the sensitivity affects certain aspects of life. The scores increase as the degree of intolerance increases[5]. Each scale contains 10 items, scored from 0 = “not a problem” to 10 = “severe or disabling problem” resulting a maximus score of 100. A score of 40-100 in chemical intolerance and symptoms, and 24-100 in Life impact predict high probability of chemical intolerance and life impact.

— *Lung function and fractional exhaled nitric oxide*

Flow-volume spirometry after cessation of long and short acting bronchodilators, and nonspecific bronchial hyperresponsiveness test (histamine or methacholine test) were performed using Medicro Pro version 4.5 (Medicro, Finland). Hyperresponsiveness was classified severe if $PD_{15}FEV_1$ in histamine challenge was ≤ 0.1mg / $PD_{20}FEV_1$ in methacholine challenge ≤ 0.15mg, respectively moderate 0.11-0.20 mg/0151-0.6 mg, mild 0.41-1.6mg/ 0.601-2.6 mg. Fractional exhaled nitric oxide was measured using an online chemiluminescence analyser (NIOX VERO,Aerocrine Ab, Sweden).

— *Skin prick tests and specific IgE*

The skin prick test panel for common environmental allergens included a negative control, a positive control (histamine), and 11 standardized environmental antigens (ALK-Abello, Hørsholm, Denmark). The results were regarded as positive if the mean wheal diameter was ≥ 3 mm and the negative control wheal was < 2 mm. In case of ≥ 2 mm wheal to negative control, specific IgE to common environmental allergens were measured using the Phadia UniCAP System (Phadia, Uppsala, Sweden). A specific IgE of < 0.35 kU/l was considered normal.

Sampling

Nasal biopsies and PBMCs were obtained from all subjects. A second set of PBMCs were obtained from 15 control individuals, following 5-6 weeks of inhaled corticosteroid use. Nasal biopsy specimens obtained from anesthetized mucosa of inferior concha were resuspended in RNALater solution and stored at -80 °C until further use. Due to the discomfort of biopsy sampling, only a single biopsy was obtained from each patient or control. As a result, the effect of medication use (biopsy sampling from the controls before and after medication use), could not be adjusted during differential expression analysis. PBMCs were isolated from venous blood (8 ml) collected into CPT tubes (BD Vacutainer CPT) via standard density gradient centrifugation.

The number of patients and samples per study groups and sample type is given in the table below.

Sample type / Individuals	AAD	APD	AND	IEI	Ctrl
**Nasal Biopsy**	n=13	n=17	n=15	n=14	n=21
**Mononuclear blood cells**	n=16	n=17	n=17	n=16	n=21
**Number of patients**	n=16	n=17	n=17	n=17	n=21

It was calculated that sample size (n= 13-21) per group is sufficiently powered for transcriptomics in this study

Considering following input values:
G_0 = 15000, non-differentially expressed genes.
$E(R_0)$ = 1, mean number of false positive genes
$\mu_1 = \log_2(2.0) = 1$, Mean difference in log-expression between patient and control groups.
$\sigma_d = \sqrt{2}\sigma = \sqrt{2}(0.40) = 0.5657$, anticipated standard deviation of the difference in log-expression between patient and control groups.
n = 13-21 per group (see number of samples per study groups and sample type above).

This input predicts, based on the method by Lee & Whitmore[6], that about 99 percent of genes that exhibit a two-fold differential expression between patient and control groups are expected to be discovered with this study design.

PBMC separation and flow cytometry

Separated plasma was aliquoted and stored at -20 °C. The extracted PBMCs were frozen in cell freezing medium (Gibco) and stored in a deep freezer (-80 °C). After thawing the samples, the number of total cells was counted (Beckman Coulter AcT/Diff), and the relative proportions of cell populations was determined by flow cytometry using surface markers. Subsets of T cells ($CD3^+$), B cells ($CD19^+$), NK cells ($CD16^+$ $CD56^+$) and monocytes ($CD14^+$) were identified. In addition, cell viability was checked and percentage of dead cells was determined (data not shown).

Cytokine profiling by Luminex assay

Frozen plasma samples were thawed, the possible precipitates were collected at 10 000 x g for 10 min at +4°C, and clear supernatants were transferred to new tubes. The samples were diluted 1:4 into the Bio-Plex sample diluent and 50 µl of the dilution was pipetted onto the plate. The soluble inflammatory markers including IL-1ra, IL-6, IL-8, IL-10, IL-12(p70) and IL-17A were measured by Bio-Plex Pro Assays (Bio-Rad) in Luminex (Bio-Rad Bio-Plex 200) system according to the provided instructions. The observed target concentrations were calculated with the help of 8 standards. And on each plate, there were three internal controls to confirm the quality between different plates.

Transcriptomics

From the nasal membrane samples (biopsy), total RNA was isolated on the RNeasy Plus Mini Kit (Qiagen, Hilden, Germany), and RNA from the white blood cells was isolated by RNA AllPrepDNA/RNA/miRNA Universal Kit (Qiagen). The amount of RNA was confirmed by Qubit and its quality was checked by a Bioanalyzer (biopsies) or by a TapeStation (PBMC samples). 100 ng of total RNA samples meeting the quality requirements were translated into complementary DNA (cDNA). Fragmented cRNA derived from nasal biopsies and blood cells was labeled with cy3 and cy5 fluorescent dyes according to the instructions of the manufacturer (Agilent Technologies, Two-Color Microarray-Based Gene Expression Analysis, Low Input Quick Amp Labeling). and finally

hybridized to Agilent microchips (G4851C, SurePrint G3 Human Gene Expression v3 8x60K). After hybridization, the slides were scanned by Agilent SureScan Dx Microarray Scanner, and the data were extracted using Agilent Feature Extraction Software.

Data analysis

We used SPSS version 25.0 (IBM Corporation, Armok, NY) for analysis of demographic and clinical parameters. Continuous measures were summarized by medians and interquartile ranges (IQR) and categorical variables by their frequencies and proportions. We analyzed the differences between the groups using the Kruskal Wallis test for continuous variables and chi-squared tests for categorical variables. Kruskal Wallis test was used also for Luminex cytokine analysis followed by Dunn's test for multiple comparisons.

For transcriptomics, gene expression changes were analyzed with *eUTOPIA* – an R-based graphical user interface composed of standard bioinformatics packages[7]. An inbuilt pipeline that is pre-configured for 2-color Agilent arrays, was used to filter out control probes, followed by log2 transformation and quantile normalization of gene chip intensities. Subsequently, batch effects introduced by dye and array during sample processing were accounted for using the *ComBat* software package[8]. Differential gene expression analysis between different test groups was performed by *Limma Model* analysis[9]. Age (biopsy and PBMC), BMI (biopsy and PBMC) and inhaled corticosteroid use (PBMC only) were used as co-factors. To adjust the variation induced by ICS in healthy controls PBMC samples collected before and after the use of ICS were included as separate samples and ICS was used as co-factor in Limma analysis. The multivariate correction of false discovery rate was performed by the *Benjamin & Hochberg* method. A minimum log2 difference of 0.58, together with a maximum adjusted p value of 0.05 was implemented as cut-off to consider a gene as significantly differentially expressed between patients and controls.

Gene-phenotype interactions between DEGs and selected clinical parameters were studied with a Pearson's correlation analysis across all patient subgroups and controls. DEGs with a correlation coefficient of $-0.5 < R > 0.5$, to at least 1 clinical parameter were selected for cluster heatmaps and for pathway enrichment analysis with Panther Gene Ontology analysis.

Clustering, heatmaps and differential expressed genes

Detrended correspondence analysis, K means clustering and principal component analysis (PCA), of either the entire transcriptome, differentially expressed genes or only those differentially expressed genes corresponding to a specific biological process, were used to visualize clustering of the subjects within this cohort. *Perseus* and *Chipster* graphical interfaces were used to generate clusters and heatmaps[10, 11].

REFERENCES

1. "Global strategy for asthma management and prevention: GINA executive summary." E.D. Bateman, S.S. Hurd, P.J. Barnes, J. Bousquet, J.M. Drazen, J.M. FitzGerald, P. Gibson, K. Ohta, P. O'Byrne, S.E. Pedersen, E. Pizzichini, S.D. Sullivan, S.E. Wenzel and H.J. Zar. Eur Respir J 2008; 31: 143-178. *Eur Respir J* **2018,** 51, (2).
2. Chung, K. F.; Wenzel, S. E.; Brozek, J. L.; Bush, A.; Castro, M.; Sterk, P. J.; Adcock, I. M.; Bateman, E. D.; Bel, E. H.; Bleecker, E. R.; Boulet, L. P.; Brightling, C.; Chanez, P.; Dahlen, S. E.; Djukanovic, R.; Frey, U.; Gaga, M.; Gibson, P.; Hamid, Q.; Jajour, N. N.; Mauad, T.; Sorkness, R. L.; Teague, W. G., International ERS/ATS guidelines on definition, evaluation and treatment of severe asthma. *Eur Respir J* **2014,** 43, (2), 343-73.

3. Vandenplas, O.; Godet, J.; Hurdubaea, L.; Rifflart, C.; Suojalehto, H.; Walusiak-Skorupa, J.; Munoz, X.; Sastre, J.; Klusackova, P.; Moore, V.; Merget, R.; Talini, D.; Kirkeleit, J.; Mason, P.; Folletti, I.; Cullinan, P.; Moscato, G.; Quirce, S.; Hoyle, J.; Sherson, D.; Kauppi, P.; Preisser, A.; Meyer, N.; de Blay, F.; European network for the, P. o. O. A. i., Severe Occupational Asthma: Insights From a Multicenter European Cohort. *J Allergy Clin Immunol Pract* **2019,** 7, (7), 2309-2318 e4.
4. Miller, C. S.; Prihoda, T. J., The Environmental Exposure and Sensitivity Inventory (EESI): a standardized approach for measuring chemical intolerances for research and clinical applications. *Toxicol Ind Health* **1999,** 15, (3-4), 370-85.
5. Nordin, S.; Andersson, L., Evaluation of a Swedish version of the Quick Environmental Exposure and Sensitivity Inventory. *Int Arch Occup Environ Health* **2010,** 83, (1), 95-104.
6. Lee, M. L.; Whitmore, G. A., Power and sample size for DNA microarray studies. *Stat Med* **2002,** 21, (23), 3543-70.
7. Marwah, V. S.; Scala, G.; Kinaret, P. A. S.; Serra, A.; Alenius, H.; Fortino, V.; Greco, D., eUTOPIA: Solution for omics data preprocessing and analysis. In Cold Spring Harbor Laboratory: 2018.
8. Leek, J. T.; Johnson, W. E.; Parker, H. S.; Jaffe, A. E.; Storey, J. D., The sva package for removing batch effects and other unwanted variation in high-throughput experiments. *Bioinformatics* **2012,** 28, (6), 882-883.
9. Ritchie, M. E.; Phipson, B.; Wu, D.; Hu, Y.; Law, C. W.; Shi, W.; Smyth, G. K., limma powers differential expression analyses for RNA-sequencing and microarray studies. *Nucleic Acids Res* **2015,** 43, (7), e47-e47.
10. Kallio, M. A.; Tuimala, J. T.; Hupponen, T.; Klemelä, P.; Gentile, M.; Scheinin, I.; Koski, M.; Käki, J.; Korpelainen, E. I., Chipster: user-friendly analysis software for microarray and other high-throughput data. *BMC Genomics* **2011,** 12, 507-507.
11. Tyanova, S.; Temu, T.; Sinitcyn, P.; Carlson, A.; Hein, M. Y.; Geiger, T.; Mann, M.; Cox, J., The Perseus computational platform for comprehensive analysis of (prote)omics data. *Nature Methods* **2016,** 13, (9), 731-740.

Supplementary figures

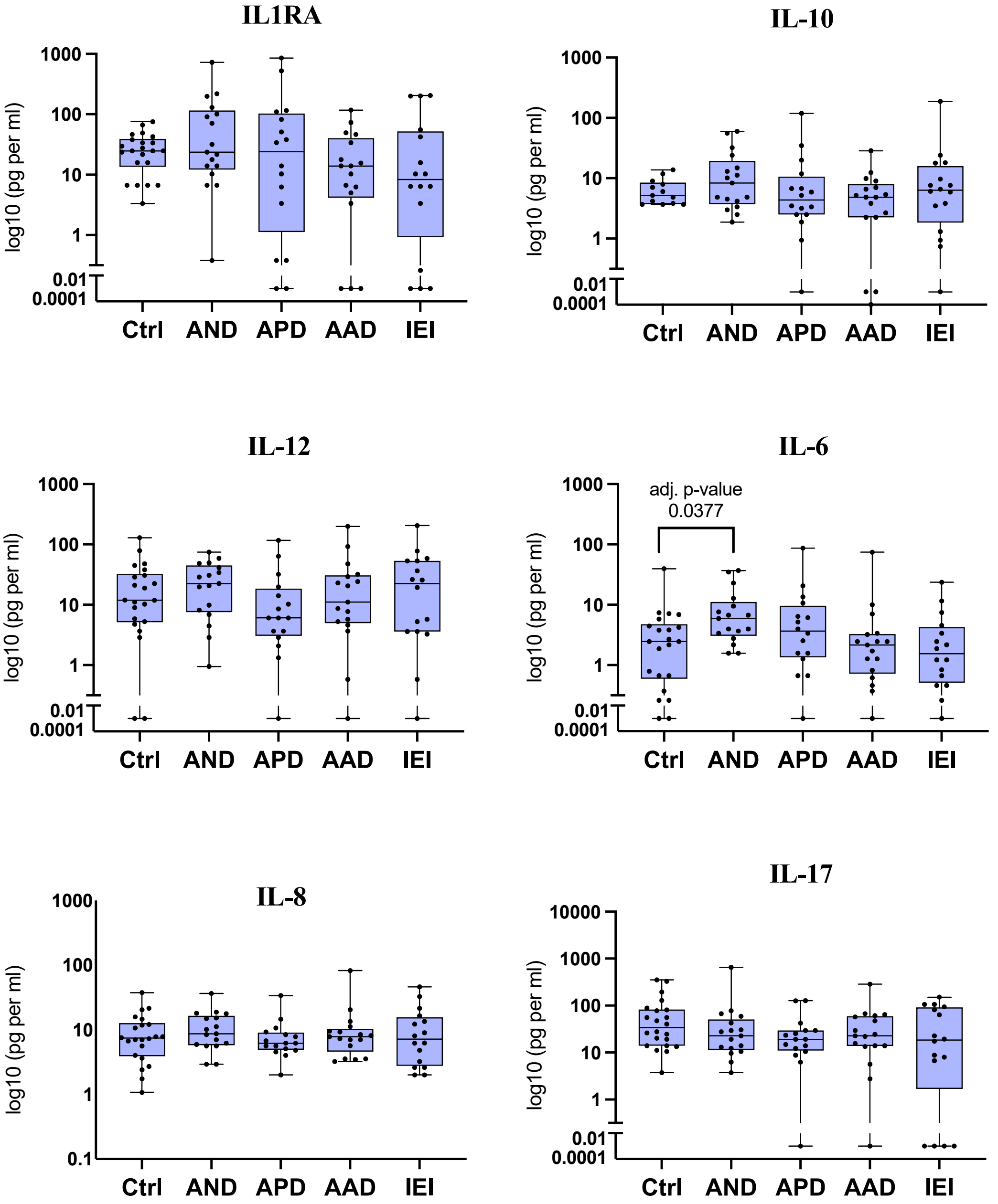

Figure S1: Profiling of selected inflammatory cytokines. AAD = asthma associated with dampness and molds, APD = asthma possibly associated with dampness and molds, AND = asthma not associated with dampness and molds, IEI = idiopathic environmental intolerance, Ctrl = Healthy control. Only IL6 was found to have a significant difference (Kruskal-Wallis test with Dunn's multiple comparison test; adjusted p value < 0.05) in abundance between patients (AND) and controls (Ctrl). Number of samples: Ctrl = 21, AND = 17, APD = 17, AAD = 16, IEI = 16.

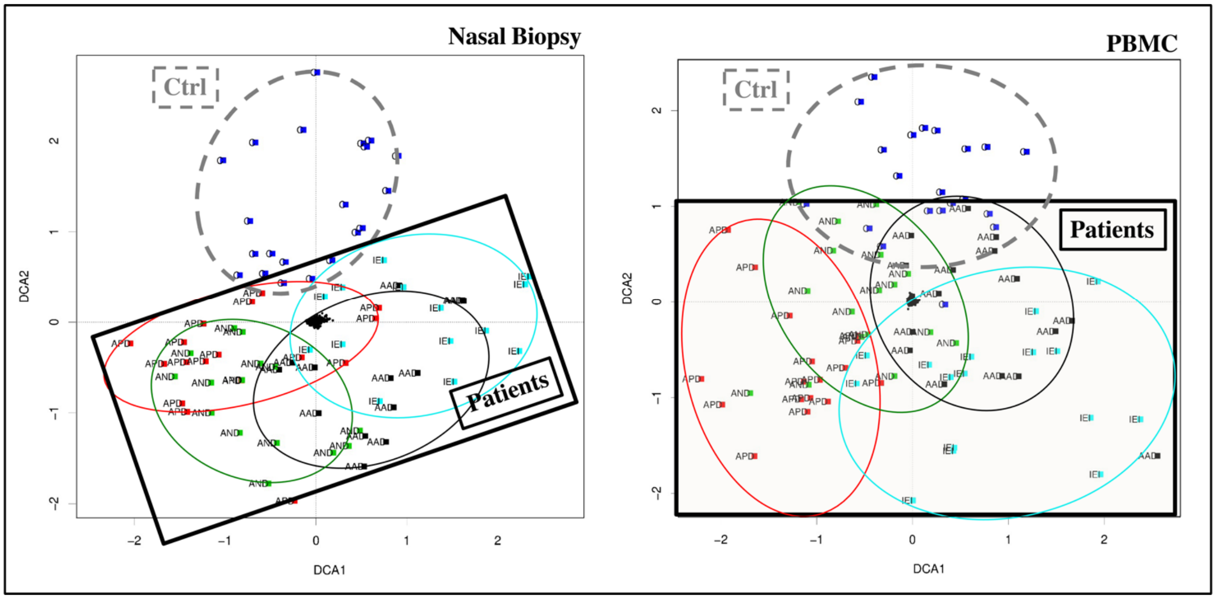

Figure S2: Detrended correspondence analysis (DCA) of batch-corrected gene expression data derived from nasal biopsies and blood cells. In total 24,288 and 21,298 genes were identified from nasal biopsy and PBMC samples, respectively. In both tissue types, a clear distinction can be seen between the gene expression profiles of controls and patients. Although the different patient subgroups can be distinguished, transcriptome heterogeneity between the different diseases appears to be minimal. AAD= asthma associated with dampness and molds, APD= asthma possibly associated with dampness and molds, AND= asthma not associated with dampness and molds, IEI= idiopathic environmental intolerance, Ctrl= Healthy control.

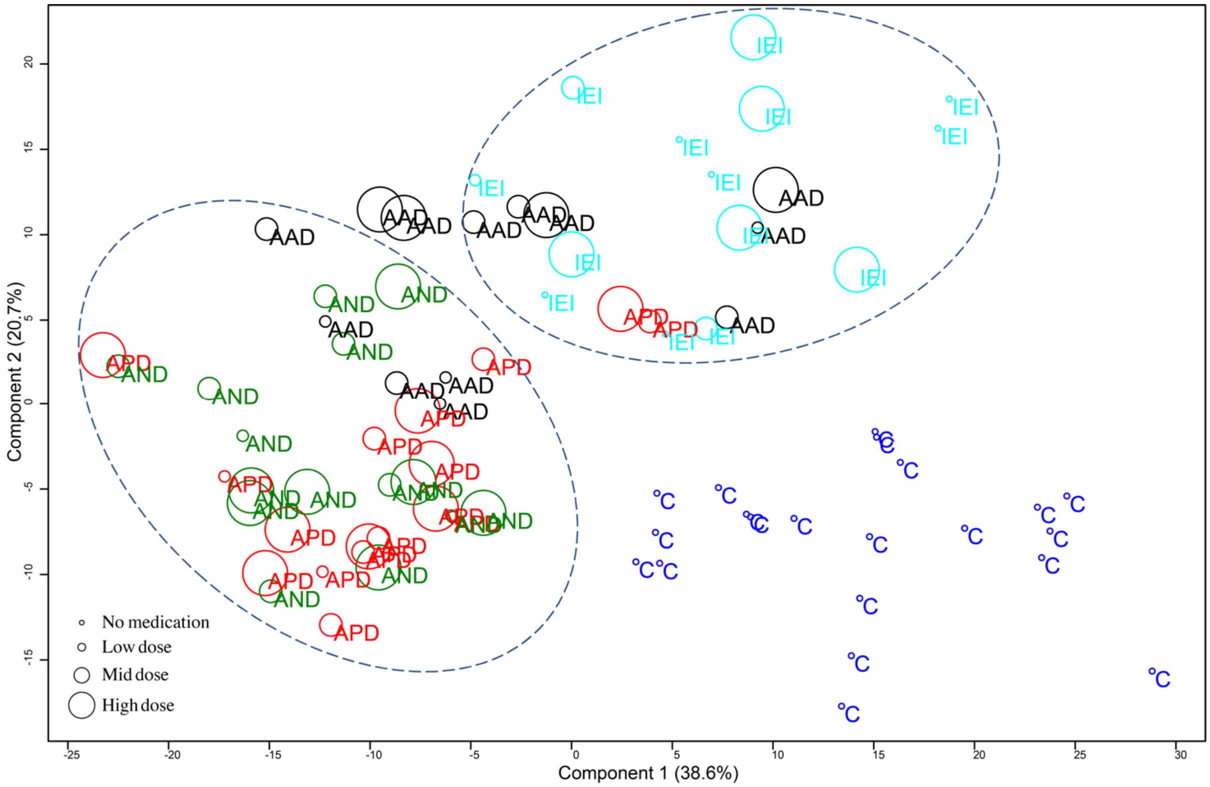

Figure S3: Principal component analysis **(**PCA) based on differential expressed genes (DEGs) from nasal biopsy. Study subgroups are abbreviated as AAD (asthma associated with dampness and molds), APD (asthma possibly associated with dampness and molds), AND (asthma not associated with dampness and molds), IEI (idiopathic environmental intolerance), C (Controls) Clear circles depict sample positions, with sizes corresponding to recent (within one month) dose of asthma medication (inhaled corticosteroids) used. No nasal biopsy samples were obtained from control subjects who had received inhaled corticosteroids, while 92% of the non-control biopsy samples were obtained from individuals undergoing treatment for asthma. Although principal components 1 and 2 explain up to 60% of the variation between these samples, no clear distinction of the different doses (no medication, low, mid and high) can be seen. Despite the lack of medicated healthy controls for the nasal biopsy tissue samples, the clear separation of IEI patients from AND and APD patients, indicate that the identified DEGs are not merely a reflection of medication use, but also disease-specific pathological mechanisms. The inhaled steroid dose in asthma patients was clinically graded according to the Global Initiative for Asthma (GINA) guidelines (GINA 2018[1]). The size of the circle is descriptive for 4 dose categories – from smallest to largest (area) being, no medication, low dose, mid dose and high dose.

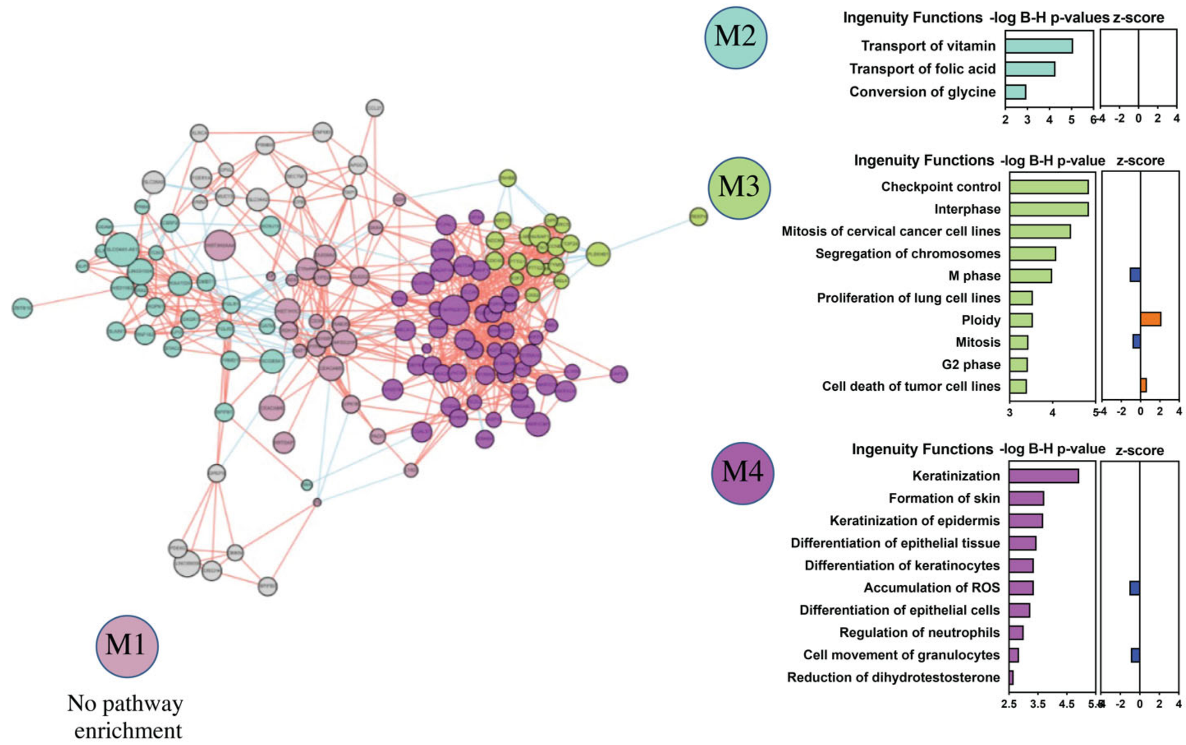

Figure S4: Prediction of the disease relevant pathways represented by differential expressed genes (DEGs) in nasal biopsy of patients with asthma that is possibly associated to damp and moldy buildings (APD). Four co-expressed gene networks (modules; M1, M2, M3 and M4) were identified from genes that are differentially expressed between *AND* patients and controls. The interactions between different inferred co-expressed gene network modules, as well as their corresponding biological processes and predicted downstream activation states are shown. Biological functions are ranked by -log of Benjamini-Hochberg corrected p values. Implemented filters: Exclude cancer pathways; Include only pathways with >= 5 differentially expressed genes). Positive (+) or negative (-) activation scores correspond to downstream activated or inhibited disease/functions, respectively. An activation Z score > |2|, is highly predictive of an activated or inhibited disease/function. No enriched biological functions were identified from the genes in module M1.

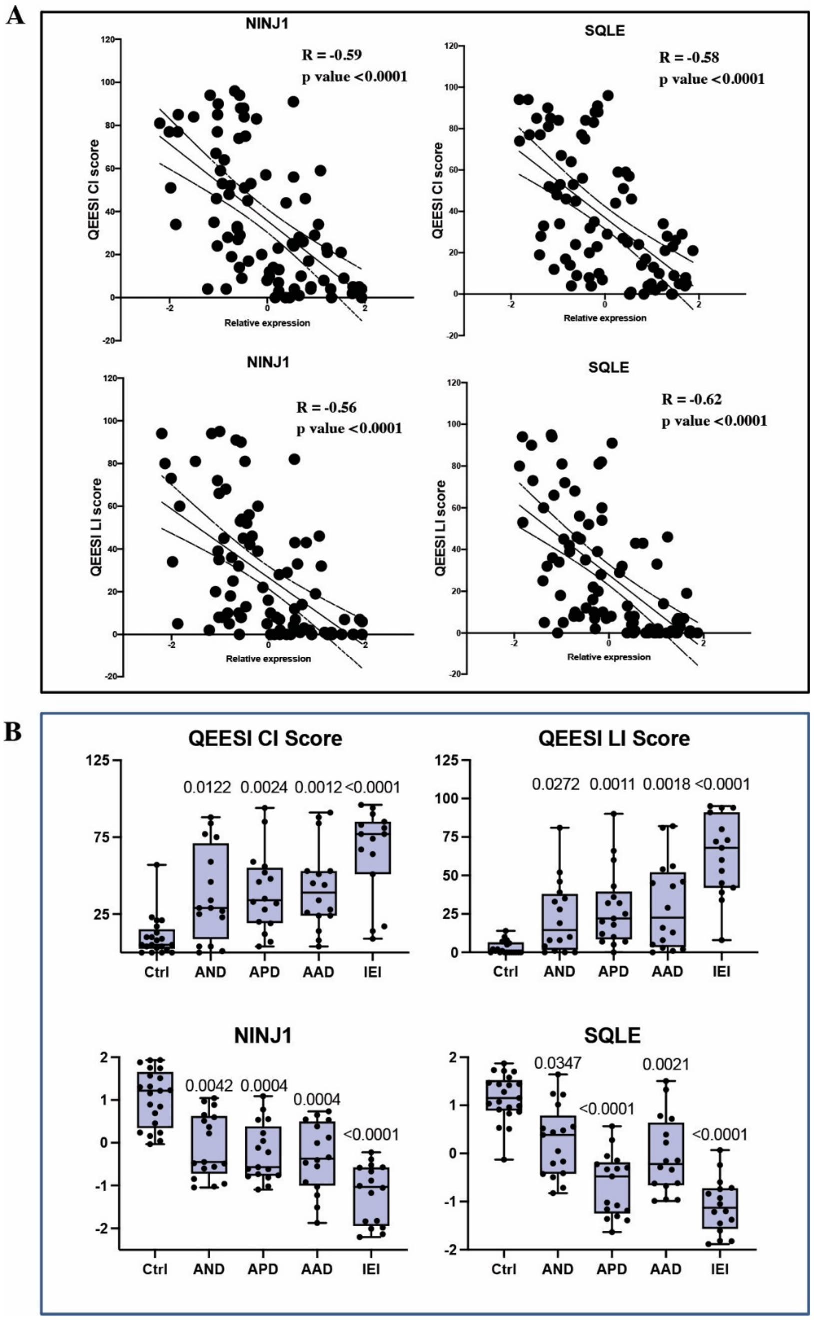

Figure S5: The top correlated genes were NINJ1 (Quick Environmental Exposure and Sensitivity Inventory life impact (QEESI-CI) score) and SQLE (QEESI- life impact (LI) score) (A). QEESI scores (top panel) and relative gene expression (bottom panel) are shown across all patient subgroups and controls (Ctrls) (B). AAD = asthma associated with dampness and molds, APD = asthma possibly associated with dampness and molds, AND = asthma not associated with dampness and molds, IEI = idiopathic environmental intolerance. Number of samples: Ctrl = 21, AND = 17, APD = 17, AAD = 16, IEI = 16. When compared with controls, QEESI scores for IEI, AAD are significantly different from controls (Kruskal-Wallis test with Dunn's post-hoc correction, p value < 0.05). Also NINJ1 and SQLE expression was significantly different for all patient/control contrasts. P value significance when compared with Ctrl, are shown above each asthma subgroup bar plot.

Supplementary tables

Table S1 (Excel file)**.** Differential expressed genes (DEGs) from all patient/control contrast in PBMC

Table S2 (Excel file)**.** Differential expressed genes (DEGs) from all patient/control contrasts in Nasal Biopsy

Table S3. Regression analyses of differential expressed genes (DEGs) and selected clinical parameters relevant to diagnosis of asthma and/or idiopathic environmental intolerance, identified 49 genes with a Pearson's correlation coefficient of -0.5 < R > 0.5, to at least 1 clinical parameter.

GeneSymbol	Log2 fold change in PBMC	adj.P.Value	Correlation to QEESI CI score (Pearson's R)	Correlation to QEESI CI score (Pearson pvalue)	Correlation to QEESI CI score (B-H adjusted Pearson pvalue)	Correlation to QEESI LI score (Pearson's R)	Correlation to QEESI LI score (Pearson pvalue)	Correlation to QEESI LI score (B-H adjusted Pearson pvalue)
NINJ1	-1.08	3.46E-03	-0.590642	3.34118E-09	5.99456E-07	-0.557529	2.98174E-08	8.38752E-07
SQLE	-0.72	2.79E-03	-0.584253	5.40045E-09	5.99456E-07	-0.618653	2.79525E-10	6.20546E-08
CDC42EP4	-0.65	3.78E-03	-0.558396	3.40526E-08	2.51992E-06	-0.536262	1.22594E-07	1.6585E-06
EAF1	-0.65	1.73E-02	-0.553217	4.83326E-08	2.68248E-06	-0.533081	1.50217E-07	1.75517E-06
HSPA5	-0.90	3.03E-03	-0.547718	6.96562E-08	3.09273E-06	-0.574864	8.73373E-09	4.84722E-07
NRIP3	-0.84	2.37E-03	-0.541625	1.03633E-07	3.39238E-06	-0.539693	9.82381E-08	1.6585E-06
PNP	-0.74	1.74E-02	-0.539334	1.20094E-07	3.39238E-06	-0.526838	2.2249E-07	2.46884E-06
MAFG	-0.62	3.45E-03	-0.539041	1.22369E-07	3.39238E-06	-0.546386	6.33111E-08	1.40551E-06
RCOR1	-0.74	2.17E-03	-0.535674	1.51625E-07	3.39238E-06	-0.557331	3.02253E-08	8.38752E-07
CTSLP8	-0.98	1.05E-02	-0.535552	1.52806E-07	3.39238E-06	-0.546536	6.2683E-08	1.40551E-06
PHLDA1	-0.87	3.32E-03	-0.533962	1.68947E-07	3.40972E-06	-0.536325	1.22101E-07	1.6585E-06
MXD1	-0.81	3.20E-03	-0.531062	2.02619E-07	3.74847E-06	-0.51856	3.70067E-07	3.11236E-06
HMGB3P1	-0.67	6.46E-04	-0.527879	2.46899E-07	4.21629E-06	-0.578727	6.5784E-09	4.84722E-07
C15orf48	-1.52	1.21E-02	-0.526636	2.66557E-07	4.22688E-06	-0.533724	1.44198E-07	1.75517E-06
LYPD3	-0.58	2.04E-02	-0.524716	2.99861E-07	4.43793E-06	-0.563809	1.92673E-08	8.38752E-07
INSIG1	-0.91	7.51E-03	-0.522296	3.47472E-07	4.82115E-06	-0.537296	1.1471E-07	1.6585E-06
NIPAL4	-1.20	3.76E-03	-0.517867	4.53722E-07	5.89953E-06	-0.512624	5.28652E-07	3.96398E-06
HOXB6	-0.82	2.44E-04	-0.516982	4.78344E-07	5.89953E-06	-0.581611	5.31092E-09	4.84722E-07
TNFRSF12A	-0.90	3.14E-02	-0.512578	6.20883E-07	7.25449E-06	-0.494847	1.4787E-06	7.42131E-06
CCL7	-0.81	1.97E-02	-0.509484	7.44115E-07	7.65393E-06	-0.488926	2.05665E-06	9.92557E-06
EHD4	-0.68	4.48E-03	-0.509368	7.49169E-07	7.65393E-06	-0.526059	2.33539E-07	2.46884E-06
CCL2	-1.15	2.87E-02	-0.508434	7.90934E-07	7.65393E-06	-0.472486	4.98035E-06	2.2564E-05
PPIF	-0.97	1.35E-02	-0.507343	8.42578E-07	7.65393E-06	-0.512402	5.35673E-07	3.96398E-06
B3GNT5	-1.19	4.57E-03	-0.506956	8.61628E-07	7.65393E-06	-0.506568	7.55405E-07	4.41316E-06
GABARAPL1	-0.85	1.01E-02	-0.50612	9.04191E-07	7.65393E-06	-0.482203	2.96927E-06	1.40063E-05
VEGFA	-0.99	2.42E-03	-0.505678	9.2747E-07	7.65393E-06	-0.494541	1.50432E-06	7.42131E-06
LDLR	-0.75	1.13E-02	-0.505232	9.51568E-07	7.65393E-06	-0.503166	9.20407E-07	5.10826E-06
IRAK2	-1.05	1.48E-02	-0.504982	9.65362E-07	7.65393E-06	-0.495652	1.41312E-06	7.29564E-06
PIM3	-0.75	2.14E-03	-0.503123	1.07369E-06	8.21936E-06	-0.537282	1.1481E-07	1.6585E-06
PRNP	-0.66	4.82E-03	-0.501242	1.19501E-06	0.000008843	-0.508596	6.70811E-07	0.00000438
JMJD1C	-0.59	4.69E-02	-0.500556	1.24234E-06	8.89647E-06	-0.481838	3.02838E-06	1.40063E-05
CTH	-0.75	2.07E-02	-0.499951	1.28558E-06	8.91885E-06	-0.544898	6.98667E-08	1.41004E-06
VDR	-0.75	1.12E-02	-0.49904	1.35335E-06	9.04519E-06	-0.500505	1.07256E-06	5.76492E-06
DNAJB5	-0.66	8.22E-03	-0.498626	1.38532E-06	9.04519E-06	-0.519486	3.49832E-07	3.10651E-06
HMOX1	-0.86	1.76E-02	-0.498081	1.42837E-06	9.06014E-06	-0.507372	7.20742E-07	4.41316E-06
EMP1	-1.21	4.36E-02	-0.494518	1.74289E-06	1.04749E-05	-0.509935	6.19969E-07	0.00000438
lnc-OR4M2-7	-0.75	7.60E-03	-0.494367	1.75756E-06	1.04749E-05	-0.513181	5.11399E-07	3.96398E-06
PPP1CB	-0.59	3.63E-03	-0.494006	1.79304E-06	1.04749E-05	-0.518187	3.7853E-07	3.11236E-06
TUFT1	-0.75	2.19E-02	-0.492791	1.91774E-06	1.09161E-05	-0.509354	6.41564E-07	0.00000438
ULBP2	-0.73	5.34E-03	-0.488493	2.42762E-06	1.34732E-05	-0.558569	2.77543E-08	8.38752E-07
PPP1R3B	-0.97	6.33E-04	-0.485339	2.88038E-06	1.55963E-05	-0.524893	2.51038E-07	2.5332E-06

GADD45G	-0.78	5.76E-04	-0.48202	3.44191E-06	1.80099E-05	-0.50657	7.55353E-07	4.41316E-06
ULBP1	-0.65	6.18E-04	-0.481769	3.48843E-06	1.80099E-05	-0.521735	3.04944E-07	2.94337E-06
SDC4	-0.71	2.67E-03	-0.480316	3.76886E-06	1.90158E-05	-0.50411	8.71485E-07	4.96076E-06
RELB	-0.69	1.20E-02	-0.474538	5.10799E-06	2.48712E-05	-0.500213	1.09066E-06	5.76492E-06
MTHFD2	-0.62	3.56E-03	-0.474367	5.15347E-06	2.48712E-05	-0.535712	1.27002E-07	1.6585E-06
EFNB2	-0.71	1.74E-02	-0.463277	9.09473E-06	4.29579E-05	-0.519785	3.43518E-07	3.10651E-06
ARG2	-0.79	1.22E-02	-0.458984	1.12712E-05	5.21284E-05	-0.509003	6.54968E-07	0.00000438
LDHA	-0.60	3.24E-03	-0.447446	1.97856E-05	8.96427E-05	-0.50664	7.52246E-07	4.41316E-06
